# Supplementary figures and images for: Reverse-zoonoses of 2009 H1N1 pandemic influenza A viruses and evolution in United States swine results in viruses with zoonotic potential
Source: PLoS Pathog. 2023 Jul 27;19(7):e1011476. doi: 10.1371/journal.ppat.1011476 (PMC10374098; doi:10.1371/journal.ppat.1011476)

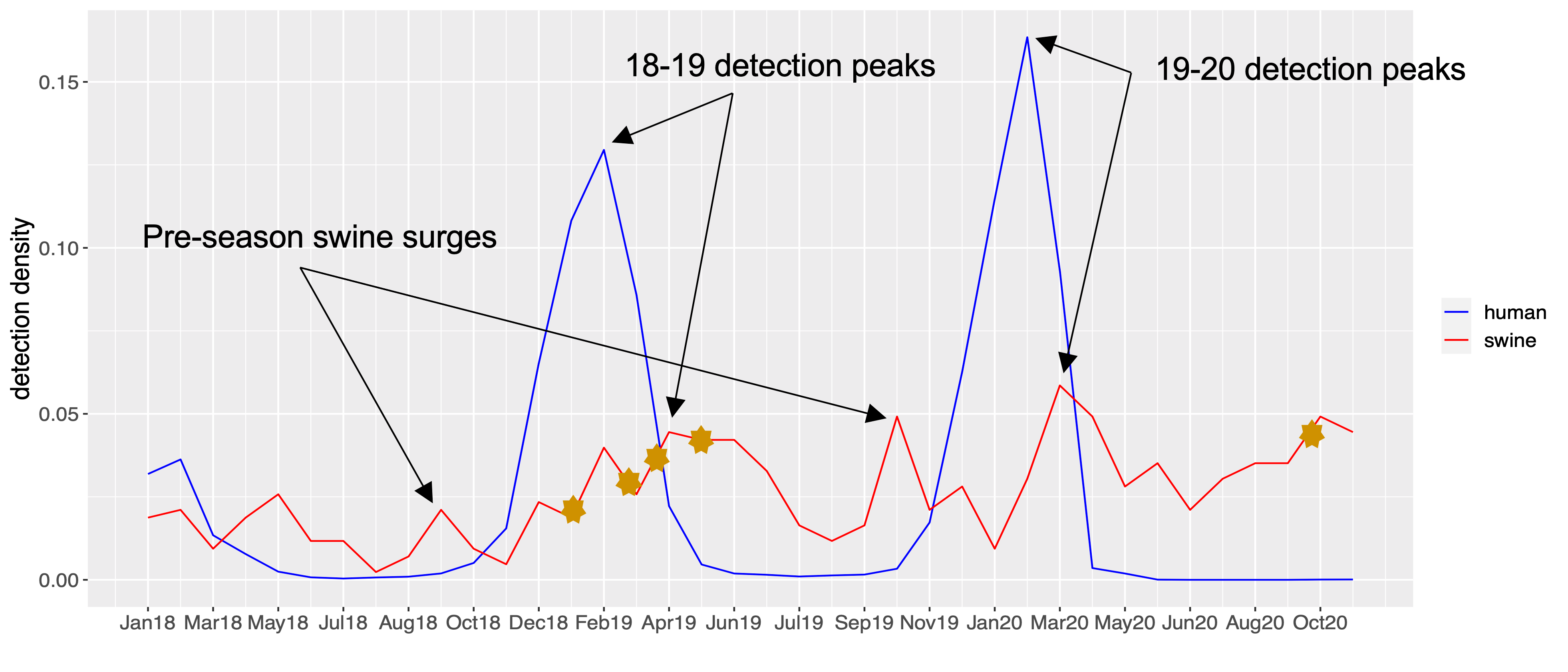

Supplement: S1 Fig — The graph captures two most severe human pdm09 seasons (line in blue) and respective increase in pdm09 circulation in US swine (line in blue). Arrows show the fall swine pdm09 detection surges as well as the 2018–19 and 2019–20 detection peaks in humans and swine. These data capture pdm09 detection frequencies throughout the 2018–19, 2019–20, and, partially, 2017–18 flu seasons. There were consistent September-October swine pdm09 detection surges that occurred prior to the start of the human flu season. These data show that in the 2018–19 and 2019–20 seasons, the human pdm09 detection peaks preceded the respective swine detection peaks, which corroborates our hypothesis that the swine pdm09 seasons were driven by human-to-swine spillovers. Variant detections during this time are marked by orange stars. (TIF) [file ppat.1011476.s001.tif]

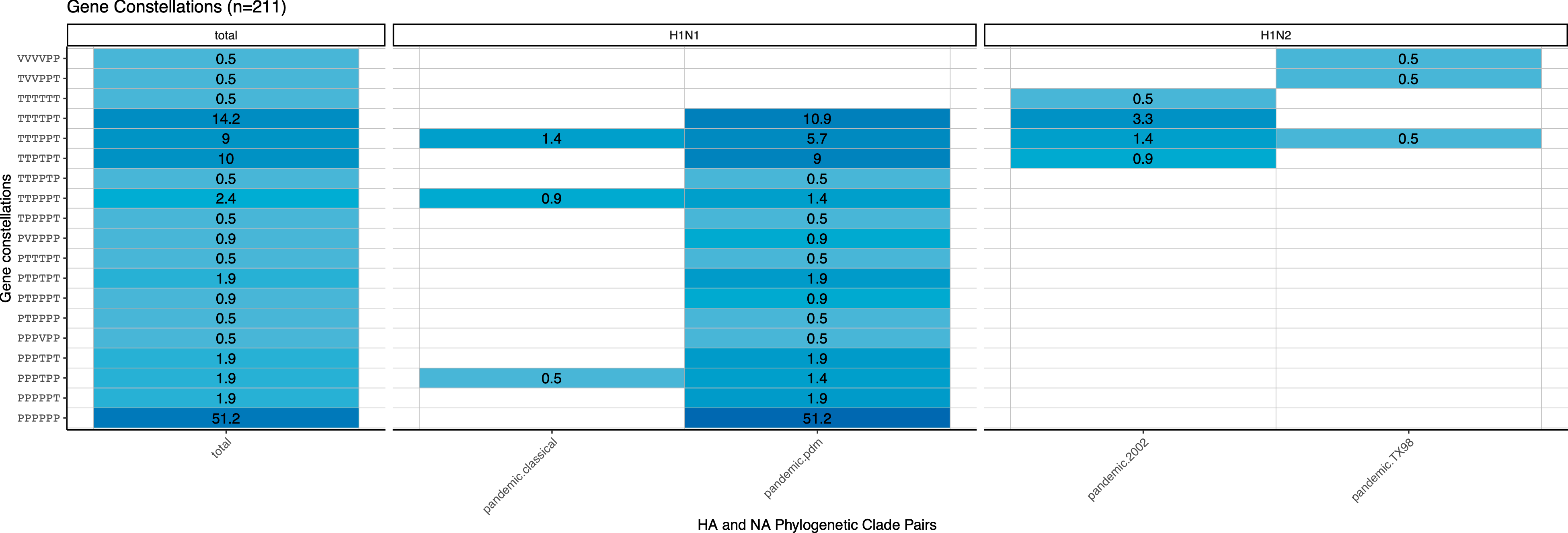

Supplement: S2 Fig — The data is based on n = 211 (2009 to 2021) whole genome sequence IAVs from the USDA influenza A in swine surveillance system. The internal genes are shown in the following order PB2-PB1-PA-NP-M-NS reflecting either the triple-reassortant (T) or H1N1pdm09 (P) evolutionary lineages, or genes derived from the live attenuated influenza vaccine (V) [57]. The 4 most common reassorted internal gene constellations were TTTTPT, TTPTPT, TTTPPT, and TTPPPT, which is consistent with 4 most common internal gene constellation among all H1N1 and H1N2 swine IAVs in the US. The data were visualized using octoFLUshow. (TIFF) [file ppat.1011476.s002.tiff]
