# Supplementary material for: Reverse-zoonoses of 2009 H1N1 pandemic influenza A viruses and evolution in United States swine results in viruses with zoonotic potential
Source: PLoS Pathog. 2023 Jul 27;19(7):e1011476. doi: 10.1371/journal.ppat.1011476 (PMC10374098; doi:10.1371/journal.ppat.1011476)
Supplement: S1 Table — Antisera were generated against Northern Hemisphere human seasonal H1N1 vaccine strains (A/California/04/2009, A/Michigan/45/2015, A/Brisbane/2/2018, A/Wisconsin/588/2019, and A/Hawaii/70/2019). The strains were selected from different persistent seasonal spillovers by generating an HA1 consensus sequence and then selecting the best-matched field isolate available at the USDA-APHIS Influenza A Virus in Swine virus repository. The internal genes are shown in the following order PB2-PB1-PA-NP-M-NS reflecting either the triple-reassortant (T) or H1N1pdm09 (P) evolutionary lineage. (DOCX) [file ppat.1011476.s005.docx]

**S1 Table. Swine H1N1pdm09 strains selected for testing against reference ferret antisera.** Antisera were generated against Northern Hemisphere human seasonal H1N1 vaccine strains (A/California/04/2009, A/Michigan/45/2015, A/Brisbane/2/2018, A/Wisconsin/588/2019, and A/Hawaii/70/2019). The strains were selected from different persistent seasonal spillovers by generating an HA1 consensus sequence and then selecting the best-matched field isolate available at the USDA-APHIS Influenza A Virus in Swine virus repository. The internal genes are shown in the following order PB2-PB1-PA-NP-M-NS reflecting either the triple-reassortant (T) or H1N1pdm09 (P) evolutionary lineage.

| Strain name | GenBank Accession | Spillover season | HA1 consensus identity | HA/NA | Internal Gene Constellation |
| --- | --- | --- | --- | --- | --- |
| A/swine/Indiana/A02525081/2021 | MW603033 | 13-14 | 99.7% | H1pdm/N1pdm | PPPPPP |
| A/swine/Colorado/A02635828/2021 | MZ485434 | 15-16 | 100% | H1pdm/N1pdm | TTTTPT |
| A/swine/Kansas/A02248038/2021 | MZ666816 | 17-18 | 99.4% | H1pdm/N1pdm | TTPTPT |
| A/swine/Iowa/A02524480/2020 | MT814305 | 18-19 | 99.7% | H1pdm/N1pdm | TTTPPT |
| A/swine/Indiana/A02635811/2021 | MZ476800 | 19-20 | 99.1% | H1pdm/N1pdm | TTTTPT |
| A/swine/Missouri/A01104146/2020 | MW579260 | 19-20 | 98.5% | H1pdm/N1pdm | PPPPPP |
