# Supplementary material for: Reverse-zoonoses of 2009 H1N1 pandemic influenza A viruses and evolution in United States swine results in viruses with zoonotic potential
Source: PLoS Pathog. 2023 Jul 27;19(7):e1011476. doi: 10.1371/journal.ppat.1011476 (PMC10374098; doi:10.1371/journal.ppat.1011476)
Supplement: S2 Table — The amino acid differences are shown relative to the human vaccine strain A/California/04/2009. (DOCX) [file ppat.1011476.s006.docx]

**S2 Table. Amino acid difference table over the HA1 subunit for the pdm09 Northern Hemisphere vaccine strains and swine pdm09 representative strains.** The amino acid differences are shown relative to the human vaccine strain A/California/04/2009.

| site | **A/California/04/2009** | **A/Michigan/45/2015** | **A/Brisbane/02/2018** | **A/Wisconsin/588/2019** | **A/Hawaii/70/2019** | A/swine/Indiana/A02525081/2021 | A/swine/Colorado/A02635828/2021 | A/swine/Kansas/A02248038/2021 | A/swine/Iowa/A02524480/2020 | A/swine/Indiana/A02635811/2021 | A/swine/Missouri/A01104146/2020 |
| --- | --- | --- | --- | --- | --- | --- | --- | --- | --- | --- | --- |
| 2 | T |  |  |  |  |  | I |  |  |  |  |
| 45 | R |  | G |  |  |  |  |  |  |  |  |
| 47 | V |  |  |  |  |  | I |  |  |  |  |
| 48 | A |  |  |  |  |  | D |  |  |  |  |
| 68 | E |  |  |  |  | G | G |  |  |  |  |
| 74 | S |  | R | R | R |  |  | R | R | R | R |
| 83 | P | S | S | S | S | S | S | S | S | S | S |
| 84 | S | N | N | N | N |  | N | N | N | N | N |
| 97 | D | N | N | N | N | N | N | N | N | N | N |
| 113 | R |  |  |  |  |  |  |  | K |  |  |
| 120 | T |  |  |  |  |  |  |  |  | A |  |
| 129 | N |  |  | D | D |  | D |  |  | D | D |
| 130 | K |  |  | N |  |  |  |  |  |  |  |
| 137 | P |  |  |  |  |  |  |  | S |  |  |
| 146 | K |  |  |  |  |  | N |  |  |  |  |
| 149 | I |  |  |  |  |  |  | V |  |  |  |
| 152 | V |  |  |  |  |  |  |  |  | I |  |
| 155 | G |  |  |  |  | E |  | E |  |  | E |
| 156 | N |  |  | K |  |  |  |  |  |  |  |
| 161 | L |  |  | I |  |  |  |  |  | I |  |
| 162 | S | N | N | N | N |  | N | N | N | N | N |
| 163 | K | Q | Q | Q | Q | Q | Q |  | Q | Q | Q |
| 164 | S |  | T | T | T |  |  | T | T | T | T |
| 173 | V |  |  |  |  |  |  |  | I |  |  |
| 183 | S |  | P | P | P |  |  | P | P | P | P |
| 185 | S | T | T | I | I | T | T | T | T | I | I |
| 187 | D |  |  |  | A |  |  |  |  |  | S |
| 189 | Q |  |  |  | E |  |  |  |  |  | E |
| 191 | I | L | L | L | L | L | L | L | L | L | L |
| 197 | T | A | A | A | A | A | A | A | A | A | A |
| 203 | S | T | T | T | T | T | T | T | T | T | T |
| 205 | R |  |  |  |  | K | K |  |  |  |  |
| 216 | I | T | T | T | T | K | S | E | T | T | T |
| 222 | D |  |  |  |  |  |  | N |  |  |  |
| 223 | Q | R | R |  |  |  |  |  |  |  |  |
| 239 | K |  |  |  |  | R |  |  |  |  |  |
| 250 | V |  |  | A |  |  |  |  |  | A |  |
| 256 | A | T | T | T | T | T | T | T | T | T | T |
| 260 | N |  |  | D | D |  |  |  |  | D | D |
| 282 | P |  | A |  |  |  |  |  |  |  |  |
| 283 | K | E | E | E | E | E | E | E | E | E | E |
| 295 | I |  | V | V | V |  |  | V | V | V | V |
| 298 | I |  | V |  |  |  |  |  |  |  |  |
| 321 | I | V | V | V | V |  | V | V | V | V | V |
| **Total aadiff** |  | **14** | **21** | **23** | **21** | **14** | **20** | **19** | **20** | **23** | **22** |
